# Comparison of robotic versus laparoscopic lateral lymph node dissection for advanced lower rectal cancer: a retrospective study at two institutions

**DOI:** 10.1007/s00464-023-09925-8

**Published:** 2023-02-09

**Authors:** Lei Zhang, Feiyu Shi, Chenhao Hu, Zhe Zhang, Junguang Liu, Ruihan Liu, Guanghui Wang, Jianqiang Tang, Junjun She

**Affiliations:** 1 Department of General Surgery, The First Affiliated Hospital, Xi’an Jiaotong University, 277 Yanta West Road, Xi’an, 710061 Shaanxi China; 2 Center for Gut Microbiome Research, Med-X Institute, The First Affiliated Hospital of Xi’an Jiaotong University, Xi’an, Shaanxi China; 3 Department of General Surgery, Peking University First Hospital, Beijing, 100034 China; 4 Department of Colorectal Surgery, National Cancer Center/National Clinical Research Center for Cancer/Cancer Hospital, Chinese Academy of Medical Sciences and Peking Union Medical College, 17 Panjiayuan Nanlu, Chaoyang District, Beijing, 100021 China

**Keywords:** Rectal cancer, Lateral lymph node dissection, Robotic surgery, Laparoscopic surgery

## Abstract

**Background:**

Lateral lymph node dissection (LLND) represents a technically challenging procedure. This study aimed to evaluate the perioperative, genitourinary functional and mid-term oncological outcomes of laparoscopic lateral lymph node dissection (LLLND) and robotic lateral lymph node dissection (RLLND) for advanced lower rectal cancer (ALRC).

**Methods:**

Between January 2015 and April 2021, consecutive patients who underwent RLLND and LLLND at two high-volume centres were enrolled. The perioperative outcomes, genitourinary function recovery and mid-term oncological outcomes of the patients were compared. A subgroup analysis of patients who underwent neoadjuvant chemoradiotherapy (nCRT) was performed.

**Results:**

A total of 205 patients were included in the analysis, with 95 in the RLLND group and 110 in the LLLND group. The patients in the RLLND group had a longer operative time, less blood loss, and more harvested internal iliac lymph nodes than did those in the LLLND group. In postoperative complication, urinary retention was less frequent in the RLLND group than in the LLLND group. Additionally, the RLLND group had better genitourinary function recovery. Similar results were also observed from the nCRT subgroup analysis. Moreover, there was no significant difference in mid-term oncological outcomes between the two groups. Further subgroup analysis indicated that the patients who underwent nCRT + LLLND/RLLND had better local control than those who underwent only LLLND/RLLND.

**Conclusions:**

RLLND is safe and feasible for ALRC and is associated with more harvested internal iliac lymph nodes and better genitourinary function recovery. NCRT combined with minimally invasive LLND could constitute an improved strategy for ALRC.

With the widespread implementation of neoadjuvant chemoradiotherapy (nCRT) before total mesorectal excision (TME) for advanced lower rectal cancer (ALRC), local recurrence (LR) rates have decreased [1]. However, recent studies have suggested that lateral lymph node metastasis (LLNM) is still a major cause of locoregional recurrence after nCRT + TME [2, 3]. In Eastern countries, TME with selective lateral lymph node dissection (LLND) is used to treat LLNM, which could cause a significant improvement in disease-free survival [4]. Thus, selective LLND combined with nCRT may be a promising approach for patients with suspected LLNM.

However, nCRT could lead to tissue oedema, fibrosis, excessive moisture, and exudates, resulting in blurry dissection planes and technical difficulty associated with the pelvic dissection [5]. Moreover, some studies have shown that LLND is associated with longer operative times, greater blood loss, and severe urogenital dysfunction [6, 7]. Some previous studies have demonstrated the feasibility of laparoscopic LLND for rectal cancer patients [8, 9]. Conventional laparoscopic rectal surgery still has some technical problems, such as limited dexterity with unstable instruments, unnatural hand–eye coordination, and flat 2-dimensional vision, which could hinder the fine and stable dissection of the TME plane [10]. Since its introduction as a new technology, robotic-assisted laparoscopic surgery has rapidly gained popularity for treating rectal cancer patients. Many studies have demonstrated that robotic surgery provides technical advantages over standard laparoscopy, such as increased freedom in the movement of instruments, enhanced dexterity, three-dimensional field of vision and more intuitive instrument manipulation, which are all ideal for complex procedures in narrow spaces, especially for LLND with pelvic autonomic nerve preservation [11]. Some retrospective studies have reported comparisons between robotic lateral lymph node dissection (RLLND) and laparoscopic lateral lymph node dissection (LLLND) [12, 13]. However, the genitourinary outcomes and oncological results of RLLND were limited, especially for those who underwent nCRT.

Thus, this study aimed to compare the clinical and genitourinary outcomes of RLLND and LLLND for ALRC. A subgroup analysis of patients who underwent nCRT + TME + LLND was further performed to evaluate the safety and feasibility of this treatment strategy.

## Materials and methods

### Study population

**Fig. 1:**
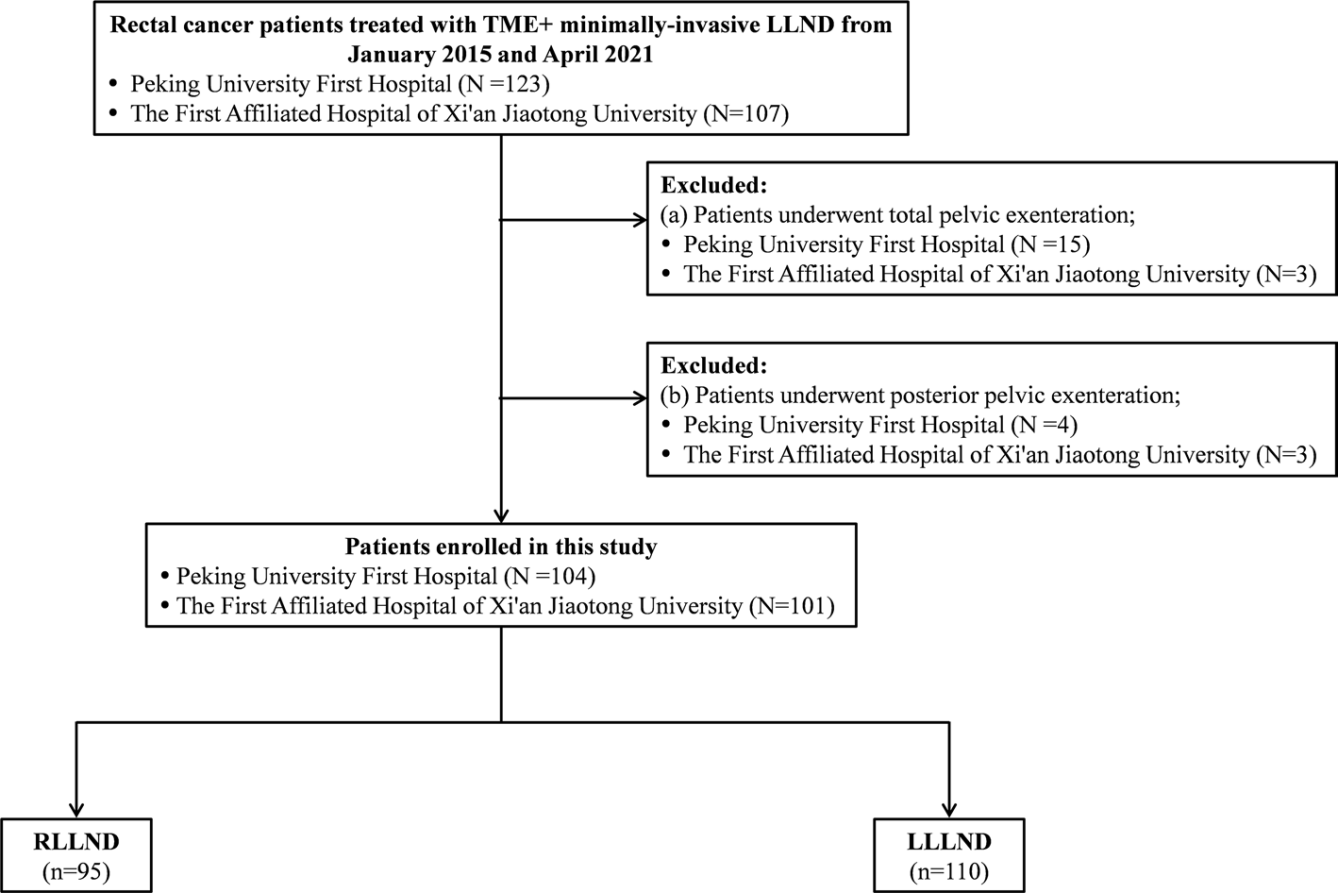
Flowchart of patient selection. *TME* total mesorectal excision, *LLND* lateral lymph node dissection, *RLLND* robotic lateral lymph node dissection, *LLLND* laparoscopic lateral lymph node dissection

From January 2015 to April 2021, a total of 230 ALRC patients with clinically suspected LLNM underwent minimally invasive LLND at The First Affiliated Hospital of Xi'an Jiaotong University and Peking University First Hospital. Patients who underwent total pelvic exenteration or posterior pelvic exenteration were excluded (*n* = 25). Thus, 205 patients with ALRC (95 patients treated with RLLND and 110 patients treated with LLLND) were included in this study (Fig. 1). Preoperative assessment, operative characteristics, postoperative complications, pathological characteristics, genitourinary outcomes, and follow-up data were collected. This study was conducted in accordance with the Declaration of Helsinki and approved by the ethics committees of the two above-mentioned institutions (2019-ZD-04).

### Treatment strategy for rectal cancer

Similar treatment strategy for rectal cancer was adopted in two institutions. All patients underwent preoperative examination consisting of imaging with computed tomography, endoscopic ultrasonography, or magnetic resonance imaging to assess tumour staging. In our institutions, nCRT was recommend to patients with clinical stage T3, T4, and/or node-positive mid and lower rectal cancers. nCRT comprised 5-fluorouracil-based concurrent chemotherapy and radiotherapy. Radiation was administered to the whole pelvis at a dose of 45 or 50 Gy in 25 fractions over 5 weeks. Curative surgery was performed 6–12 weeks after the completion of nCRT. However, some patients who refused nCRT were treated with TME and LLND. LLND was recommended to rectal cancer patients with positive lateral lymph nodes (LLNs) were suspected or persistently enlarged LLNs ≥ 5 mm after nCRT. A suspicious LLN was defined as a lymph node that was enlarged by more than 5 mm in the short-axis diameter with irregular borders on imaging assessment. The measurements were made with imaging workstation electronic callipers. Unilateral or bilateral LLND was performed depending on the preoperative imaging findings or intraoperative findings. Only when both pelvic sidewalls had clinical suspected LLNM, bilateral LLND was considered. All the patients provided informed consent before surgery. Postoperative adjuvant chemotherapy was given to stage III or stage II patients with high-risk factors based on the pathological results.

### Operative technique

In two institutions, all the surgeries were performed by the same team of experienced surgeons with rich experience in robotic and laparoscopic surgery, respectively. Our technique for RLLND has been reported in our previous publications [14]. The sequence of surgical steps for both the robotic and laparoscopic procedures was identical, except for the port position. After TME was completed, LLND was performed. LLND involved complete removal of the lateral pelvic lymph nodes in the fatty tissues, including the internal iliac nodes (#263), the obturator nodes (#283), and/or common iliac nodes (#273), and/or external iliac nodes (#293), with preservation of the bilateral hypogastric nerve and pelvic nerve plexus.

### Postoperative complications and mortality

Postoperative complications were stratified according to the Clavien–Dindo classification system [15]. Complications of grade ≥ III were defined as severe complications. Each patient was assessed for C-D grading by two experienced surgeons, and divergences were resolved by discussion. Urinary retention was defined as residual urine volume > 50 ml after the foley catheter was removed [11]. Anastomotic leakage or anastomotic bleeding was diagnosed based on clinical signs and symptoms, such as abdominal pain or fever, with faecal or haemorrhagic material in the pelvic drain or peritonitis. Operative mortality was defined as postoperative death from any cause within 30 days or during the same hospitalization.

### Assessment of genitourinary functional outcome

All patients were requested to complete the Chinese version of the International Prostatic Symptom Score (IPSS) questionnaire at baseline (before treatment) and at intervals up to 1 year after surgery [16]. This questionnaire is based on seven symptoms related to urinary function with a five-scale system that can be answered on a scale from ‘never’ (score 0) to ‘almost always’ (score 5). The symptoms assessed were incomplete emptying, frequency, intermittency, urgency, weak stream, straining, and nocturia. The 5-item version of the International Index of Erectile Function (IIEF-5) was used to assess male sexual function [17]. Because some patients in our study did not have regular sexual activity, we used a telephonic method to follow-up patients’ postoperative changes in erectile function. Patients who were followed up for less than 1 year, were unwilling to participate, or had incomplete functional surveys were excluded.

### Oncological outcomes

The postoperative follow-up protocol was the same for both groups. Patients were scheduled for follow-up visits every 3 months for the first 2 years, then every 6 months thereafter until 5 years, and every year thereafter. The monitoring programme for recurrence comprised a physical examination, serum CEA and CA19-9 levels, colonoscopy, chest/abdominal/pelvic CT, or MRI. The deadline for follow-up of patients was February 2022, and the median follow-up period was 38.0 months. The overall survival (OS) time was calculated from operation time to death or follow-up deadline. Relapse-free survival (RFS) was defined as survival without LR or distant metastases. LR was defined as recurrence within the pelvic cavity, including the inguinal lymph nodes, which required imaging or pathological evaluation.

### Statistical analysis

All statistical analyses were conducted using the Statistical Package for the Social Sciences for Windows, version 26 (IBM Corp., Armonk, NY, USA). Categorical variables were compared using the *χ*^2^ test or Fisher’s exact test (for one or more cells with expected values < 5). Continuous variables were compared using Student’s *t* test or the Wilcoxon rank-sum test. The Kaplan–Meier method was used to evaluate patient prognosis, with groups compared by the log-rank test. *P* values less than 0.05 were considered statistically significant. All statistical tests were 2-sided.

## Results

A total of 205 patients from two institutions were collected. There were 95 patients in the robotic group (54 males; mean age of 60.4 years) and 110 patients in the laparoscopic group (74 males; mean age of 58.3 years). There were no significant between-group differences in age, sex, body mass index (BMI), American Society of Anesthesiologists (ASA) status, tumour location from the anal verge, preoperative chemoradiotherapy, history of previous abdominal operations, clinical S stage or institution (Table 1). Furthermore, the patients who had undergone nCRT were further included as a subgroup analysis and were stratified into RLLND (*n* = 37) or LLLND (*n* = 44) groups. In the nCRT cohort groups, the baseline characteristics of the RLLND and LLLND groups were also balanced (Table 1).

**Table 1:** Baseline clinicopathologic characteristics of patients with locally advanced low rectal cancer undergoing LLND

Variable	Overall cohort			nCRT cohort		
	RLLND (*n* = 95)	LLLND (*n* = 110)	*P* value	RLLND (*n* = 37)	LLLND (*n* = 44)	*P* value
Age, yrs, mean ± SD	60.4 ± 12.5	58.3 ± 11.6	0.425	58.1 ± 12.8	57.6 ± 7.6	0.883
Male sex, no. (%)	54(56.8)	74(67.3)	0.124	11(29.7)	21(47.7)	0.099
BMI (kg/m^2^), mean ± SD	22.4 ± 3.5	23.8 ± 3.6	0.214	21.7 ± 3.4	23.4 ± 2.7	0.119
ASA score,no. (%)	–	–	0.299	–	–	0.505
I-II	82(86.3)	89(80.9)		33(89.2)	37(84.1)	–
III	13(13.7)	21(19.1)		4(10.8)	7(15.9)	–
Tumour distance from anal verge, cm, mean ± SD	4.6 ± 2.4	4.1 ± 1.8	0.484	4.2 ± 2.0	5.5 ± 2.1	0.518
Preoperative chemoradiation therapy, no. (%)	37(38.9)	44(40.0)	0.878	–	–	–
Previous abdominal surgery, no. (%)	11(11.6)	7(6.4)	0.188	3(8.1)	4(9.1)	0.875
cSstage	–	–	0.587	–	–	0.411
I	0	0		0	0	–
II	17(17.9)	23(20.9)		5(13.5)	9(20.5)	–
III	78(82.1)	87(79.1)		32(86.5)	35(79.5)	–
Institution	–	–	0.146	–	–	0.081
The First Affiliated Hospital of Xi'an Jiaotong University	52(54.7)	49(44.5)		24(64.9)	20(45.5)	–
Peking University First Hospital	43(45.3)	61(55.5)	–	13(35.1)	24(54.5)	–

*LLND* lateral lymph node dissection, *nCRT* neoadjuvant chemoradiotherapy, *RLLND* robotic lateral lymph node dissection, *LLLND* laparoscopic lateral lymph node dissection, *BMI* body mass index, *ASA* American Society of Anesthesiologists

Table 2 shows the operative outcomes. The type of procedure did not significantly differ between the groups. In the overall cohort groups, the median operating time for RLLND was significantly longer [255 min (robotic) vs. 220 min (laparoscopic); *P* = 0.001]. In the nCRT cohort groups, the median operating time for RLLND was still longer [275 min (robotic) vs. 255 min (laparoscopic); *P* = 0.007]. There was no difference in the time of unilateral LLND between the RLLND group and the LLLND group. Whether for the overall cohort groups or the nCRT cohort groups, the estimated blood loss was significantly less for the robotic group [80 ml (robotic) vs. 110 ml (laparoscopic); *P* = 0.027] and [100 ml (robotic) vs. 130 ml (laparoscopic); *P* = 0.030]. Regarding the index of postoperative recovery, there was no difference between the overall cohort and the nCRT cohort except for the time of Foley catheter removal [3 days (robotic) vs. 4 days (laparoscopic); *P* = 0.024] and [3 days (robotic) vs. 5 days (laparoscopic); *P* = 0.008].

**Table 2:** Perioperative outcomes and postoperative recovery of patients with locally advanced low rectal cancer undergoing LLND

Variable	Overall cohort			nCRT cohort		
	RLLND (*n* = 95)	LLLND (*n* = 110)	*P* value	RLLND (*n* = 37)	LLLND (*n* = 44)	*P* value
Type of operation, no. (%)	–	–	0.144	–	–	0.416
LAR	58(61.1)	54(49.1)		20(54.1)	25(56.8)	
ISR	23(24.2)	29(26.4)		9(24.3)	6(13.6)	
APR	14(14.7)	27(24.5)		8(21.6)	13(29.5)	
LLND, no. (%)	–	–	0.135	–	–	0.083
Unilateral (left/right)	48(50.5)	67(60.9)		21(56.8)	33(75.0)	
Bilateral	47(49.5)	43(39.1)		16(43.2)	11(25.0)	
Operation time (min), median (range)	255(160–420)	220(110–500)	0.001	275(185–405)	255(165–500)	0.007
Unilateral LLND time (min), median (range)	35(19–49)	42(17–63)	0.354	39(20–45)	45(23–63)	0.127
Estimated blood loss (mL), median (range)	80(20–400)	110(10–500)	0.027	100(20–400)	130(40–500)	0.030
Days to first flatus (days), median (range)	2(1–6)	3.5(2–9)	0.178	2(1–5)	3.5(2–7)	0.336
Days to soft diet (days), median (range)	5(2–16)	6(2–25)	0.156	5(3–16)	5(2–25)	0.241
Postoperative hospital stay (days), median (range)	9(3–25)	11(6–59)	0.221	10(5–25)	10.5(6–59)	0.556
Postoperative urinary catheter removal date (days), median (range)	3(1–14)	4(2–24)	0.024	3(2–14)	5(2–24)	0.008
Postoperative α-blocker use, no, (%)	22(23.2)	36(32.7)	0.129	10(27.0)	14(31.8)	0.638

*LLND* lateral lymph node dissection, *nCRT* neoadjuvant chemoradiotherapy, *RLLND* robotic lateral lymph node dissection, *LLLND* laparoscopic lateral lymph node dissection, *LAR* low anterior resection, *ISR* intersphincteric resection, *APR* abdominoperineal resection

Table 3 shows the pathological outcomes. In the overall cohort groups, 28 patients in the RLLND group (29.5%) and 41 patients in the LLLND group (37.3%) conformed to LLNM (*P* = 0.239). There was no significant difference in tumour size, grade of differentiation, pathological stage, number of harvested total lymph nodes, or number of harvested unilateral LLNs. However, the median number of harvested #263 was significantly higher in the RLLND group compared with the other groups [3 (robotic) vs. 2 (laparoscopic); *P* = 0.037]. In the nCRT cohort groups, there was no difference in any pathological outcomes between the two groups.

**Table 3:** Postoperative pathologic outcomes of patients with locally advanced low rectal cancer undergoing LLND

Variable	Overall cohort			nCRT cohort		
	RLLND (*n* = 95)	LLLND (*n* = 110)	*P* value	RLLND (*n* = 37)	LLLND (*n* = 44)	*P* value
The size of tumour (cm),mean ± SD	4.5 ± 1.7	4.4 ± 1.8	0.399	4.0 ± 1.3	3.6 ± 1.8	0.417
Grade of differentiation,no.(%)	–	–	0.103	–	–	0.807
Well or moderately differentiated	58(61.1)	79(71.8)		26(70.3)	32(72.7)	–
Poorly differentiated/mucinous carcinoma/signet ring cell	37(38.9)	31(28.2)		11(29.7)	12(27.3)	–
p/ypT stage, no.(%)	–	–	0.421	–	–	0.269
T1/T2	20(21.1)	32(29.1)		7(18.9)	13(29.5)	–
T3/T4	75(78.9)	78(70.9)		30(81.1)	31(70.5)	–
p/ypN stage, no.(%)	–	–	0.552	–	–	0.273
N0	48(50.5)	51(46.4)		14(37.8)	22(50.0)	
N1/N2	47(49.5)	59(53.6)		23(62.2)	22(50.0)	
Harvested no.of lymph nodes, median (range)	24(11–66)	23(8–70)	0.314	17(11–37)	15(8–27)	0.283
Harvested no.of unilateral lateral lymph nodes, median (range)	8(1–25)	6(1–19)	0.203	7(1–16)	6(1–19)	0.424
Harvested no.of #263, median (range)	3(1–14)	2(1–17)	0.037	2(1–6)	2(1–10)	0.722
Harvested no.of #273, median (range)	1(1–7)	1(1–10)	0.745	1(1–6)	1(1–3)	0.729
Harvested no.of #283, median (range)	3(1–13)	2(1–9)	0.105	2(1–5)	2(1–4)	0.517
Harvested no.of #293, median (range)	2(1–13)	2(1–6)	0.435	1(1–5)	1(1–3)	0.414
Lateral lymph node metastasis, no.(%)	28(29.5)	41(37.3)	0.239	9(24.3)	9(20.5)	0.676

*LLND* lateral lymph node dissection, *nCRT* neoadjuvant chemoradiotherapy, *RLLND* robotic lateral lymph node dissection, *LLLND* laparoscopic lateral lymph node dissection, *#263* internal iliac nodes, *#283* obturator nodes, *#273* common iliac nodes, *#293* external iliac nodes

Table 4 shows the postoperative morbidity and mortality. In the overall cohort groups, the rates of overall complications [18.9% (robotic) vs. 27.3% (laparoscopic); *P* = 0.160] and major complications [5.3% (robotic) vs. 6.4% (laparoscopic); *P* = 0.738] were similar between the two groups. The types of complications were also comparable. However, the rate of urinary retention was significantly lower in the robotic group than in the laparoscopic group [7.4% (robotic) vs. 17.3% (laparoscopic); *P* = 0.034]. No operative mortality occurred in either group. In the nCRT cohort groups, no significant differences were identified in the types of postoperative complications.

**Table 4:** Postoperative morbidity and mortality of patients with locally advanced low rectal cancer undergoing LLND

Variable	Overall cohort			nCRT cohort		
	RLLND (*n* = 95)	LLLND (*n* = 110)	*P* value	RLLND (*n* = 37)	LLLND (*n* = 44)	*P* value
Any complication, no.(%)	18(18.9)	30(27.3)	0.160	8(21.6)	14(31.8)	0.304
Anastomotic leak	1(1.2)	3(3.6)	0.323	1(3.4)	1(3.2)	0.962
Anastomotic bleeding	2(2.5)	1(1.2)	0.560	1(3.4)	0(0.0)	0.297
Small bowel obstruction	1(1.1)	2(1.8)	1.000	0(0.0)	1(2.3)	1.000
Urine retention^a^	7(7.4)	19(17.3)	0.034	3(8.1)	9(20.5)	0.119
Urinary infection	2(2.1)	3(2.7)	1.000	1(2.9)	1(2.3)	1.000
Pelvic infection	3(3.2)	2(1.8)	0.665	1(2.9)	2(7.7)	1.000
Wound infection	2(2.1)	2(1.8)	1.000	1(2.9)	1(2.3)	1.000
Pulmonary infection	2(2.1)	1(0.9)	0.597	1(2.9)	0(0.0)	0.457
Peroneal nerve palsy	1(1.1)	2(1.8)	1.000	1(2.9)	1(2.3)	1.000
Major complications^§^	5(5.3)	7(6.4)	0.738	3(8.1)	3(6.8)	0.825
Operative mortality^╋^	0 (0.0)	0 (0.0)	N/A	0 (0.0)	0 (0.0)	N/A

^a^residual urine volume > 50 ml was considered urinary retention

^§^Clavien-Dindo classification 3 or more

^╋^In-hospital or 30-day mortality

**Fig. 2:**
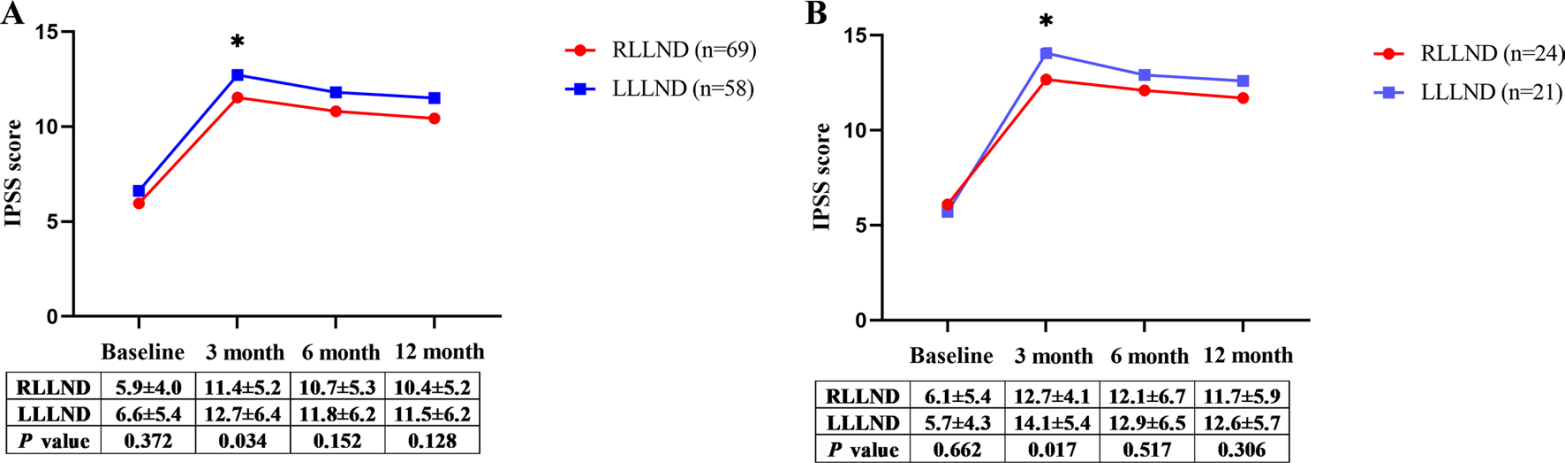
In the **A** (overall cohort) and **B** (nCRT cohort), change in total International Prostatic Symptom Score from baseline to 3, 6, and 12 months after surgery *nCRT* neoadjuvant chemoradiotherapy, *IPSS* International Prostatic Symptom Score, *RLLND* robotic lateral lymph node dissection, *LLLND* laparoscopic lateral lymph node dissection, *✱* significant differences between groups

*LLND* lateral lymph node dissection, *nCRT* neoadjuvant chemoradiotherapy, *RLLND* robotic lateral lymph node dissection, *LLLND* laparoscopic lateral lymph node dissection,

A total of 127 patients completed postoperative urinary functional evaluation. The mean IPSS scores at baseline and 3 months, 6 months and 12 months after surgery are shown in Fig. 2. There was no significant difference in the preoperative total IPSS scores between the RLLND and LLLND groups. After surgery, the IPSS scores increased in both groups. There was a significant intergroup difference at 3 months after surgery in both the overall cohort (11.4 ± 5.2 (robotic) vs. 12.7 ± 6.4 (laparoscopic); *P* = 0.034) and the nCRT cohort (12.7 ± 4.1 (robotic) vs. 14.1 ± 5.4 (laparoscopic); *P* = 0.017). The IPSS scores were still higher in the LLLND group than in the RLLND group at 6 and 12 months after surgery, but the difference between the two groups was not significantly different.

The distributions of erectile function for each group are shown in Table 5. A total of 103 patients completed postoperative sexual function evaluation. In the overall cohort groups, before surgical resection, 11 patients in the RLLND group and 12 patients in the LLLND group had moderate-to-severe erectile dysfunction. There was no intergroup difference. The proportion of patients with moderate-to-severe erectile dysfunction was significantly higher in the LLLND group at 3 months after surgery than in the RLLND group [67.4% (robotic) vs. 86.7% (laparoscopic); *P* = 0.019]. In the nCRT cohort groups, before surgical resection, 2 patients in the RLLND group and 2 patients in the LLLND group had moderate-to-severe erectile dysfunction. However, no significant intergroup differences were observed at 3, 6 and 12 months after surgery. This could be due to the relatively small sample size.

**Table 5:** Changes of erectile function of male patients undergoing LLND

Variable	Overall cohort			nCRT cohort		
	RLLND (*n* = 43)	LLLND (*n* = 60)	*P* value	RLLND (*n* = 11)	LLLND (*n* = 19)	*P* value
Baseline	–	–	0.502	–	–	0.552
No or mild ED	32(74.4)	48(80.0)		9(81.8)	17(89.5)	–
Moderate-to-severe ED	11(25.6)	12(20.0)		2(18.2)	2(10.5)	–
3 months	–	–	0.019	–	–	0.236
No or mild ED	14(32.6)	8(13.3)		3(27.3)	2(10.5)	–
Moderate-to-severe ED	29(67.4)	52(86.7)		8(72.7)	17(89.5)	–
6 months	–	–	0.084	–	–	0.346
No or mild ED	20(46.5)	18(30.0)		6(54.5)	7(36.8)	–
Moderate-to-severe ED	23(53.5)	42(70.0)		5(45.5)	12(63.2)	–
12 months	–	–	0.138	–	–	0.757
No or mild ED	25(58.1)	26(45.0)	–	7(63.6)	11(57.9)	–
Moderate-to-severe ED	18(41.9)	34(55.0)	–	4(36.4)	8(42.1)	–

*LLND* lateral lymph node dissection, *nCRT* neoadjuvant chemoradiotherapy, *RLLND* robotic lateral lymph node dissection, *LLLND* laparoscopic lateral lymph node dissection, *ED* erectile dysfunction

**Fig. 3:**
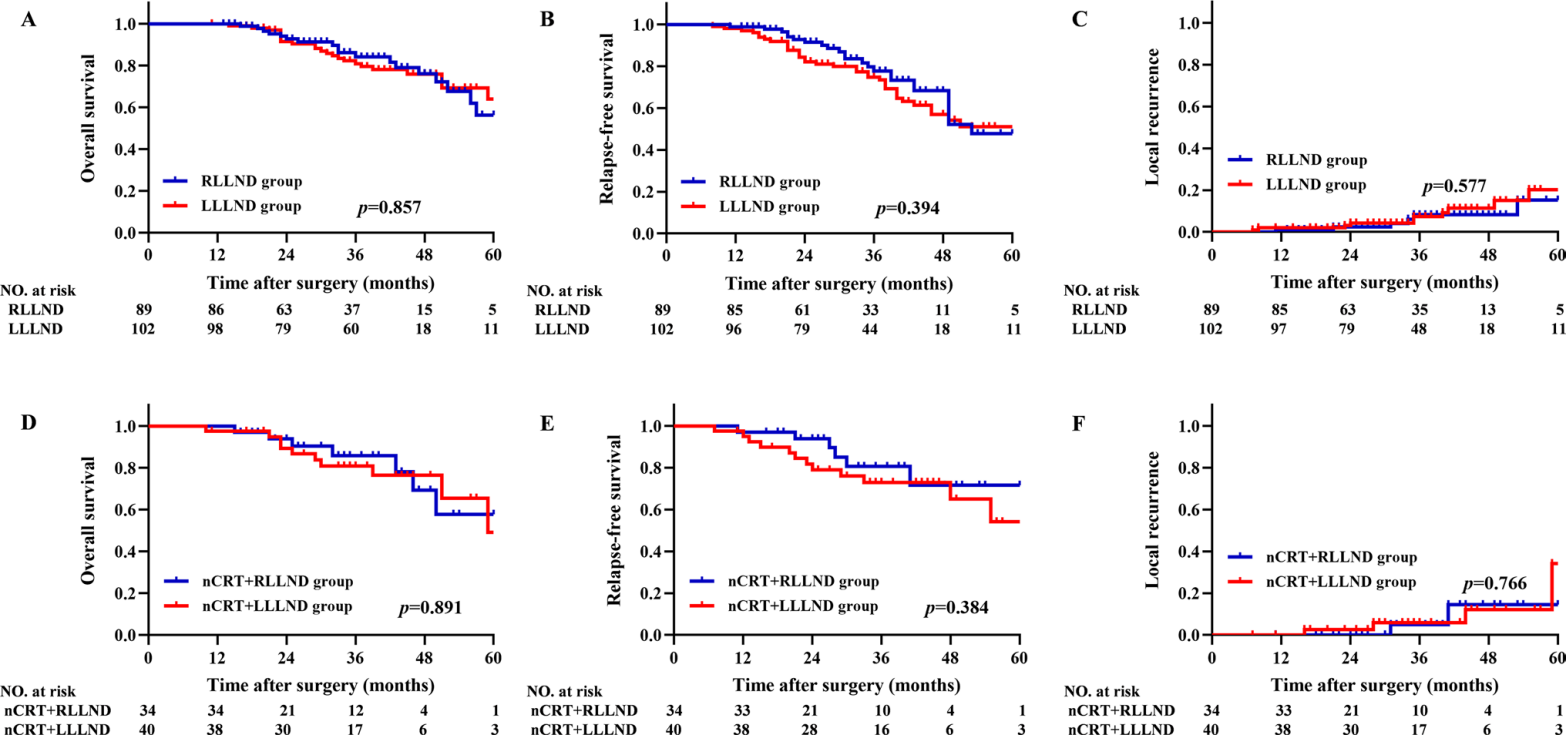
In the overall cohort, **A** (overall survival), **B** (relapse-free survival), and **C** (Local recurrence) rate of robotic (*n* = 89) and laparoscopic (*n* = 102) lateral lymph node dissection. In the nCRT cohort, **D** (overall survival), **E** (relapse-free survival) and **F** (Local recurrence) rate of robotic (*n* = 34) and laparoscopic (*n* = 40) lateral lymph node dissection. *RLLND* robotic lateral lymph node dissection, *LLLND* laparoscopic lateral lymph node dissection, *nCRT* neoadjuvant chemoradiotherapy

In the overall cohort groups, the median follow-up duration was 38.0 (range 11.0–83.0) months in 191 patients. The 3-year OS rates were 84.1 and 80.9% in the RLLND and LLLND groups, respectively (*P* = 0.857) (Fig. 3A). The 3-year RFS rates were 77.7 and 74.9% in the RLLND and LLLND groups, respectively (*P* = 0.394) (Fig. 3B), and the 3-year LR rates were 8.2 and 7.3% in the RLLND and LLLND groups, respectively (*P* = 0.577) (Fig. 3C). In RLLND group, there were 6 recurrences, including 4 developed anastomotic recurrence and 2 developed perineal recurrence. In LLLND group, there were 10 recurrences, including 3 developed anastomotic recurrence, 3 developed presacral recurrence, 2 developed anterior recurrence and 2 developed perineal recurrence.

In the nCRT cohort groups, the median follow-up duration was 36.0 (range 10.0–76.0) months in 74 patients. The 3-year OS rates were 85.8 and 80.9% in the RLLND and LLLND groups, respectively (*P* = 0.891) (Fig. 3D), the 3-year RFS rates were 80.6 and 73.0% in the RLLND and LLLND groups, respectively (*P* = 0.384) (Fig. 3E), and the 3-year LR rates were 5.0 and 5.9% in the RLLND and LLLND groups, respectively (*p* = 0.766) (Fig. 3F). In nCRT + RLLND group, there were 3 recurrences, including 2 developed anastomotic recurrence and 1 developed perineal recurrence. In nCRT + LLLND group, there were 5 recurrences, including 2 developed anastomotic recurrence, 2 developed anterior recurrence and 1 developed perineal recurrence.

**Fig. 4:**
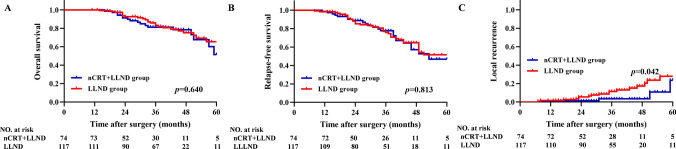
Kaplan–Meier curves of **A** (overall survival), **B** (relapse-free survival) and **C** (local recurrence) in patients with rectal cancer who only underwent LLND (*n* = 117) or nCRT + LLND (*n* = 74). *LLND* lateral lymph node dissection, *nCRT* neoadjuvant chemoradiotherapy

The prognosis of the LLND group and nCRT + LLND group was further compared. The 3-year OS rates were 81.3 and 83.3% in the nCRT + LLND and LLND groups, respectively (*P* = 0.640) (Fig. 4A), the 3-year RFS rates were 77.7 and 75.9% in the nCRT + LLND and LLND groups, respectively (*P* = 0.813) (Fig. 4B), and the 3-year LR rates were 3.4 and 11.4% in the nCRT + LLND and LLND groups, respectively (*P* = 0.042) (Fig. 4C).

## Discussion

This study compared the short-term outcomes, functional outcomes and mid-term oncological outcomes between laparoscopic and robotic LLND for local ALRC. Our study was the first large-scale study to evaluate the dynamic changes in postoperative genitourinary function from robotic LLND. Primarily, we found that patients who underwent RLLND showed significantly better genitourinary functional recovery, especially < 3 months after surgery. Although laparoscopic or robotic LLND was safe and feasible and resulted in acceptable postoperative morbidity and oncological outcomes, robotic surgery was associated with a lower amount of estimated blood loss and a lower incidence of urinary retention than the laparoscopic approach. Furthermore, our subgroup results indicated that the above advantages of robotic surgery remained valid for patients who underwent nCRT.

To the best of the authors’ knowledge, the present study was the first to evaluate the efficiency of LLND by comparing the harvested LLNs of different regions. It is well known that the quality of lymph node dissection should be evaluated by the number of retrieved lymph nodes [18]. Ozawa et al. [19] reported that the number of harvested LLN metastases had a significant association with prognosis: patients with three or more LLN metastases had worse survival than did those with only one or two LLN metastases. A high-volume study demonstrated that #263 and #283 were major metastatic regions of the lateral pelvic wall and that #273 and #293 metastases were associated with a worse prognosis [20]. Thus, it is necessary to divide LLNs into different regions according to anatomic location. In comparison to the number of dissected lymph nodes of the lateral pelvic wall, the total number of harvested unilateral LLNs was higher in the RLLND group than in the LLLND group [8 (robotic) vs. 6 (laparoscopic); *P* = 0.203], but the difference was not significant. The number of harvested #263 was significantly higher in the RLLND group than in the LLLND group [3 (robotic) vs. 2 (laparoscopic); *P* = 0.037]. Therefore, the technical advantage of robots may be reflected in the dissection of some specific areas. Site #263 is located between the ureterohypogastric nerve fascia and the medial aspect of the vesicohypogastric fascia adjacent to the internal iliac artery and the autonomic nerves, which means it is highly technically difficult to work in this region. The robotic system provided the wristed instruments enable ambidextrous capability, intuitive manipulation and a stable 3D high-definition image in a narrow pelvic cavity. It was thereby easier to identify lymphatic tissue and protect the autonomic nerve plexus between the pelvic parietal fascia and the major pelvic structures. Technically, the greatest challenge of LLND is dissecting the distal internal iliac nodes (#263D), which are located very deep in the pelvic space. The robotic approach can facilitate access to the depths of the pelvis due to more operator-controlled retraction, better optics and instrument precision, which could achieve thorough dissection of #263D. However, in the nCRT groups, the difference in the number of harvested #263 was not significant [2 (robotic) vs. 2 (laparoscopic); *P* = 0.722]. This may be due to the decreased number of lymph nodes resulting from neoadjuvant therapy, leading to the difference not being obvious. In our study, the numbers of harvested #273 and #293 were similar between the 2 groups. This may be because the laparoscopic techniques are competent for the procedure, which is not too difficult in dissecting #273 and #293. With respect to our study’s treatment strategy, only the enlarged #273 and #293 detected preoperatively and during the operation were dissected. This strategy may lead to the number of #273 and #293 analysed being too small to draw an accurate conclusion.

The overall postoperative complication rate was 23.4%, with 18.9% in the robotic group and 27.3% in the laparoscopic group, which were not significantly different. The incidence of grade 3 or 4 postoperative complications was also similar between the robotic and laparoscopic groups (5.3% and 6.4%). However, our study found a significantly lower incidence of urinary retention in the robotic group than in the laparoscopic group (7.4% vs. 17.3%, *P* = 0.034). Kagawa et al. [11] reported an 8% incidence of urinary retention in the robot-assisted LLND group, which is consistent with our findings. In our study, patients who underwent RLLND 3 months after surgery showed better genitourinary functional recovery. Although no significant difference in urogenital function was shown 12 months postoperatively, this represents an acceptable functional result overall. This phenomenon may be attributable to the extent of lymph node dissection and to the use of nerve-sparing techniques. When ipsilateral nodal involvement with no clinical contralateral nodal involvement occurs, the surgical fields for LLND are reduced to cover only the affected hemipelvis to minimize the possibility of autonomic nerve injuries. Unilateral complete preservation of the pelvic autonomic nerve alone can result in good urinary function and erection ability. Akasu et al. [21] reported that LLND with autonomic nerve preservation is associated with a low rate of urinary dysfunction. Moreover, compared with laparoscopic surgery, robotic surgery can achieve better autonomic nerve protection. In the dissection of #263D, the inferior vesical artery and vein, the internal pudendal artery and vein and sacral nerve should be fully exposed to complete the dissection of #263D. The three-dimensional magnetic views of the robotic system could provide good visual identification of these structures to avoid unnecessary intraoperative injury. When patients receive neoadjuvant therapy, a variety of factors, such as tissue oedema, fibrosis, and enlarged lymph nodes invading the pelvic autonomic nerve, can greatly increase the risk of postoperative urogenital dysfunction. Robotic surgery allows for gentle and cautious handling of nervous tissues through proper traction and countertraction. These advantages of the robotic system help surgeons better use nerve-sparing techniques, which might contribute to less urogenital dysfunction.

Nevertheless, controversy remains regarding the treatment of lateral pelvic sidewalls in patients with local ALRC. Increasing evidence suggests that nCRT is not sufficient as a stand-alone therapy to eradicate LLNM [22, 23]. Malakorn et al. [24] demonstrated that a postneoadjuvant chemoradiation lateral pelvic lymph node size ≥ 5 mm was strongly associated with pathologic positivity. Thus, in our study, patients who underwent nCRT were further analysed. First, regarding the results of selective LLND, LLNM was found in 18/81 (22.2%) patients underwent nCRT in this study. Second, in terms of prognosis, our study showed that the 3-year LR rate was significantly lower in the nCRT + LLND group than in the only LLND group (3.4 vs. 11.4%, *P* = 0.042). Akiyoshi et al. [3] reported that the combination of nCRT and LLND can significantly improve the local control and even survival of patients with LLNM compared with TME + LLND and that LLNM is a regional disease that can be equally cured as mesorectal lymph node metastasis. Thus, compared with stand-alone therapy, only LLND or nCRT, nCRT combined with selective LLND might be a more reasonable and effective approach to enhance the effect of local control.

There were several limitations to this study. First, this was a retrospective study conducted at two institutions; some inherent selection bias may exist. Second, the sample size in the present study might not be large enough, especially the number of patients who underwent nCRT, to draw accurate conclusions. Third, the follow-up period of this study was not long enough to adequately evaluate the long-term oncological outcomes. Therefore, further prospective, randomized trials with long-term follow-up surveys are needed to address these issues. However, the authors believe that this report provides useful results that may be useful to individualize treatment strategies for locally ALRC patients.

## Conclusion

RLLND is a safe and feasible technique and resulted in better recovery of urogenital function than did LLLND for ALRC patients. nCRT + TME + RLLND may be considered a promising approach in the treatment of rectal cancer.

## Abbreviations

RLLND: Robotic lateral lymph node dissection
LLLND: Laparoscopic lateral lymph node dissection
LLND: Lateral lymph node dissection
TME: Total mesorectal excision
nCRT: Neoadjuvant chemoradiotherapy
ALRC: Advanced lower rectal cancer
LR: Local recurrence
LLNM: Lateral lymph node metastasis
LLN: Lateral lymph node
#263: Internal iliac nodes
#263D: Distal internal iliac nodes
#283: Obturator nodes
#273: Common iliac nodes
#293: External iliac nodes
IPSS: International Prostatic Symptom Score
IIEF-5: The 5-item version of the International Index of Erectile Function
BMI: Body mass index
ASA: American Society of Anesthesiologists
OS: Overall survival
RFS: Relapse-free survival
